# Interleukin-24 Regulates T Cell Activity in Patients With Colorectal Adenocarcinoma

**DOI:** 10.3389/fonc.2019.01401

**Published:** 2019-12-10

**Authors:** Yang Zhang, Ye Liu, Yuechao Xu

**Affiliations:** ^1^Department of Gastrointestinal Surgery, The First Hospital of Jilin University, Changchun, China; ^2^Intensive Care Unit, 964th Hospital of PLA, Changchun, China

**Keywords:** colorectal cancer, interleukin-24, T lymphocytes, immunoregulation, anti-tumor

## Abstract

Interleukin (IL)-24 plays a potential anti-tumor activity in colorectal cancer in a dose-dependent manner. However, the immunoregulatory role of IL-24 to peripheral and tumor-infiltrating T cell function in colorectal cancer was not fully elucidated. In this study, twenty-nine colorectal adenocarcinoma patients and fifteen healthy individuals were enrolled. IL-24 expression and IL-24 receptor (IL-20R1, IL-20R2, and IL-22R1) mRNA relative level was measured by ELISA and real-time PCR, respectively. CD4^+^ and CD8^+^ T cells were purified from peripheral bloods and cancer specimens, and were stimulated with low (10 ng/ml) and high (100 ng/ml) concentration of recombinant IL-24. CD4^+^ T cells activity was assessed by measurement of Th cell percentage, transcriptional factors, and cytokine production. CD8^+^ T cells activity was evaluated by investigation of cytotoxic molecules, target cell death, and interferon-γ (IFN-γ) secretion. IL-24 was decreasingly expressed in both peripheral bloods and cancer tissues in colorectal adenocarcinoma patients. However, IL-20R1 and IL-20R2 was comparable between healthy controls and colorectal adenocarcinoma patients. Low concentration of IL-24 suppressed CD4^+^ T cell proliferation. In contrast, high concentration of IL-24 not only promoted CD4^+^ T cell proliferation, but also enhanced CD4^+^ T cell activity, which mainly presented as up-regulation of Th1/Th17 frequency, T-bet/RORγt mRNA, and IFN-γ/IL-17 production but down-regulation of Treg percentage, FoxP3 mRNA, and IL-10/IL-35 secretion. Moreover, high concentration of IL-24 also increased perforin and granzyme B expression in CD8^+^ T cells, and elevated cytolytic and non-cytolytic activity of CD8^+^ T cells, which presented as induction of target cell death and elevation of IFN-γ expression. However, low concentration of IL-24 did not affect bioactivity of CD8^+^ T cells. The current data indicated that IL-24 might regulate T cell function in a dose-dependent manner. High-concentration of IL-24 might promote anti-tumor immune responses in development novel therapeutic approaches to colorectal adenocarcinoma.

## Introduction

Colorectal cancer is the third most commonly diagnosed malignancy and the fourth leading cause of tumor-related death all over the world, accounting for ~1.4 million newly diagnosed cases and almost 0.7 million deaths annually (1–3). Although colorectal cancer incidence and mortality rates have been stabilizing or declining in a number of high human development index countries, the rapid increases in incidence and mortality of colorectal cancer are observed in medium or low income countries (4) and in patients <40 years old (5, 6). Moreover, most cases of colorectal cancer develop slowly over several years through adenocarcinoma sequence despite strong hereditary components (7). The therapeutic approaches for colorectal cancer are surgery, neoadjuvant radiotherapy (for rectal cancer), and adjuvant chemotherapy (for stage III/IV and high-risk stage II colon cancer) (7). However, the 5-year survival rate for colorectal cancer patients ranges from more than 90% in stage I patients to slightly higher than 10% in stage IV patients (8). Thus, it is pivotal to better understand the biological and immunological mechanism for colorectal cancer progression and clinical relevant insights for management of the disease.

Interleukin (IL)-24, which is also called melanoma differentiation associated gene-7, is a IL-20 cytokine family member and is expressed primarily in T cells and marcophages (9, 10). IL-24 receptor belongs to type II cytokine receptor family, and consists of two heterodimeric receptor complexes, IL-20R1/IL-20R2 and IL-22R1/IL-20R2 (11). IL-24 is a potential anti-tumor agent and affects a broad array of cancers, which selectively inhibits tumor cell growth, invasion, metastasis, and angiogensis, induces cancer-selective apoptosis, stimulates anti-cancer immune response, sensitizes cancer call to therapies (12, 13). Importantly, IL-24 is a dose-dependent cytokine, and different concentrations of IL-24 may present completely contrary bioactivity. Low concentration of IL-24 promotes inflammatory cytokine expression, leading to the proinflammatory response. In contrast, high concentration of IL-24 strongly induces apoptosis of cancer cells (12, 14). IL-24 expression was remarkably correlated with histological differentiation, but inversely correlated with the degree of lymph node involvement in rectal cancer (15), which played an important anti-tumor role for colon cancer therapy (16, 17) and even reversed multidrug resistance to chemotherapy in human colorectal cancer cells (18). However, few studies focused on the regulatory activity of IL-24 to immune cell function in colorectal cancers. Thus, we hypothesized that IL-24 also modulates CD4^+^ and CD8^+^ T cell activity in colorectal adenocarcinoma patients in a dose-dependent manner. To test this possibility, the effects of different concentrations of recombinant IL-24 stimulation on CD4^+^ and CD8^+^ T cells from colorectal adenocarcinoma patients were investigated in cell culture system *in vitro*.

## Materials and Methods

### Enrolled Subjects

The current study protocol was approved by the Ethics Committee of The First Hospital of Jilin University, and written consent form was obtained from each enrolled subjects. A total of 24 colorectal adenocarcinoma patients, who were hospitalized and underwent surgery between July 2107 and December 2018 in Department of Gastrointestinal Surgery of The First Hospital of Jilin University, were enrolled in this study. Blood samples, fresh colorectal adenocarcinoma specimens and patient-matched normal tissues were obtained. No patients received chemotherapy, radiotherapy, or immunomodulatory therapy prior to surgery. The tumor-node-metastasis (TNM) stages were evaluated according to the American Joint Committee on Cancer/Union for International Cancer Control TNM classification (7th ed.). For healthy controls, 15 healthy individuals with matched age and sex ratio, who received routine medical health test in our hospital, were also enrolled for blood sampling. The clinical characteristics of enrolled subjects were shown in Table 1.

**Table 1:** Clinical characteristics of enrolled subjects.

	**Colorectal adenocarcinoma**	**Healthy control**
Cases (*n*)	29	15
Sex (male/female)	19/10	10/5
Age (years)	55.7 ± 10.1	56.2 ± 7.4
Tumor site (right-sided/left-sided/transverse)	14/9/6	Not available
Differentiation (well/moderate/poor)	7/18/4	Not available
TNM stage (I/II/III/IV)	12/14/3/0	Not available

### Peripheral Blood Mononuclear Cells (PBMC) and Tumor-Infiltrating Lymphocytes (TIL) Isolation

PBMCs were isolated using Ficoll-Hypaque (Sigma, St. Louis, MO, USA) density gradient centrifugation from anticoagulant peripheral bloods of all enrolled subjects. TILs were isolated from tissue specimens, which were passed through 70 μm-pore strainers. Cells were treated with Collagenase D (0.5 mg/ml) at 37°C for 30 min, and then were resuspended in 44% Percoll in RPMI 1640 (*vol*/*vol*). The resuspended cells were layered over 56% Percoll in PBS (*vol*/*vol*), and were centrifuged at 850 × *g* for 30 min. The interphase, which contained TILs, was collected and washed twice. TILs were cultured in RPMI 1640 supplemented with 10% fetal bovine serum at a concentration of 10^6^/ml.

### CD4^+^ and CD8^+^ T Cells Purification

CD4^+^ and CD8^+^ T cells were purified from PBMCs and TILs using human CD4^+^ T Cell Isolation Kit (Miltenyi, Bergisch Galdbach, Germany) and human CD8^+^ T Cell Isolation Kit (Miltenyi), respectively, according to the instructions from manufacturer. The purity of enriched cells was more than 95% as determined by flow cytometry analysis.

### Cell Culture

Purified CD4^+^ T cells or CD8^+^ T cells were stimulation with recombinant human IL-24 (R&D System, Minneapolis, MN, USA; final concentration: 10 ng/ml or 100 ng/ml) for 24 h in the presence of anti-CD3/CD28 (eBioscience, San Diego, CA, USA; final concentration: 1 μg/ml). In certain experiments, 5 × 10^4^ of IL-24 stimulated CD8^+^ T cells from HLA-A2 restricted patients were co-cultured in direct contact and in parallel in indirect contact system with 2.5 × 10^5^ of colorectal adenocarcinoma cell line CACO-2, which was also HLA-A2 restricted (19), for 48 h in the presence of anti-CD3/CD28 (Invitrogen eBioscience; final concentration: 1 μg/ml). Briefly, in direct contact co-culture system, CD8^+^ T cells and CACO-2 cells were mixed directly in a cell culture plate. In indirect contact co-culture system, CD8^+^ T cells and CACO-2 cells were separated by a 0.4 μm-pore membrane in a Transwell culture plate (Corning, Corning, NY, USA), which allowed the passage of soluble factors only (20). Cells and supernatants were harvested for further experiments.

### Enzyme Linked Immunosorbent Assay (ELISA)

The cytokine expression in the plasma or cultured supernatants was measured using commercial ELISA kits (R&D System) according to the instructions from manufacturer.

### Real-Time Polymerase Chain Reaction (PCR)

Total RNA was isolated from cells or tissues using RNeasy Minikit (Qiagen, Hilden, Germany) according to the instructions from manufacturer. RNA was reversely transcribed using PrimeScript RT Master Mix (TaKaRa, Beijing, China) with random hexamers. Real-time PCR was performed using TB Green Premix *Taq* (TaKaRa). The relative gene expression was quantified by 2^−ΔΔ*CT*^ method using ABI7500 System Sequence Detection Software (Applied Biosystems, Foster, CA, USA). To normalize the absolute quantification according to a single reference gene, kinetic PCR reactions has to be performed for β-actin on all experimental samples and the relative abundance values are calculated for internal control as well as for the target gene. For each target gene sample, the relative abundance value obtained is divided by the value derived from the control sequence (β-actin) in the corresponding target gene. The normalized values for different samples can then directly be compared. The primer sequences were shown in Table 2.

**Table 2:** Primer sequences for real-time PCR.

**Primer**	**Sequence**
IL-24 forward	5′-GGR TTG TTC CCT GTG TCA TT-3′
IL-24 reverse	5′-GCG CTG CTT AAA GAA TGA CT-3′
IL-20R1 forward	5′-TCA AAC AGA ACG TGG TCC CAG TG-3′
IL-20R1 reverse	5′-TCC GAG ATA TTG AGG GTG ATA AAG-3′
IL-20R2 forward	5′-GCT GGT GTC CAC TCA CTG AAG GT-3′
IL-20R2 reverse	5′-TCT GTC TGG CTG AAG GCG CTG TA-3′
IL-22R1 forward	5′-CCC CAG ACA ACG GTC TAC AGC AT-3′
IL-22R1 reverse	5′-GGG TCA GGC CGA AGA ACT CAT AT-3′
T-bet forward	5′-CGG CTG CAT ATC GTT GAG GT-3′
T-bet reverse	5′-GTC CCC ATT GGC ATT CCT C-3′
ROR-γt forward	5′-AGT CGG AAG GCA AGA TCA GA-3′
ROR-γt reverse	5′-CAA GAG AGG TTC TGG GCA AG-3′
FoxP3 forward	5′-CCT CCC CCA TCA TAT CCT TT-3′
FoxP3 reverse	5′-TTG GGG TTT GTG TTG AGT GA-3′
β-actin forward	5′-AGC GGG AAA TCG TGC GTG-3′
β-actin reverse	5′-CAG GGT ACA TGG TGG TGC C-3′

### Cellular Proliferation Assay

Cellular proliferation was measured using Cell Counting Kit-8 (CCK-8; Beyotime, Wuhan, Hubei Province, China) according to the instructions from manufacturer.

### Flow Cytometry

CD4^+^ T cells were stained with anti-CD3-APC Cy7 (clone SK7; BD Pharmingen, San Jose, CA, USA), anti-CD4-PerCP (clone S3.5; Invitrogen eBioscience) along with anti-interferon-γ (IFN-γ)-FITC (clone 4S.B3; Invitrogen eBioscience; for intracellular staining), anti-FoxP3-PE (clone 150D/E4; Invitrogen eBioscience; for intracellular staining) and anti-IL-17A-APC (clone eBio64DEC17; Invitrogen eBioscience; for intracellular staining). CD8^+^ T cells were stained with anti-CD3-APC Cy7 (clone SK7; BD Pharmingen), anti-CD8-APC (clone 17D8; Invitrogen eBioscience) along with anti-perforin-PE (clone delta G9; Invitrogen eBioscience; for intracellular staining), anti-granzyme B-PE (clone GB11; Invitrogen eBioscience; for intracellular staining), or anti-Fas ligand (FasL, CD178)-PE (clone NOK-1; Invitrogen eBioscience; for surface staining), respectively. Acquisitions were performed using Cell Quest Pro Software (BD Biosciences Immunocytometry Systems, San Jose, CA, USA) in a FACS Aira II analyzer (BD Biosciences Immunocytometry Systems). Data were analyzed using FlowJo Software Version 8.4.2 for Windows (Tree Star, Ashland, OR, USA).

### Cytotoxicity of Target CACO-2 Cells

The cytotoxicity of target CACO-2 cells was assessed by measurement of lactate dehydrogenase (LDH) expression in cultured supernatants at the end of incubation period using LDH Cytotoxicity Assay Kit (Beyotime) according to the instructions from manufacturer. A low-level LDH control was represented as LDH expression in CACO-2 cells, while a high-level LDH control was represented as LDH expression in Triton X-100-treated CACO-2 cells. The percentage of target cell death was calculated using the following equation: (experimental value – low-level LDH control)/(high-level LDH control – low-level LDH control) × 100%.

### Statistical Analysis

Data were analyzed using SPSS 21.0 Version for Windows (SPSS, Chicago, IL, USA). Shapiro-Wilk test was used for normal distribution assay. The parameters following normal and skewed distribution were shown in Table S1. Variables following normal distribution were presented as mean ± standard deviation. Student's *t*-test was used for comparison between two groups. One-way ANOVA followed by Tukey test for multiple comparison was used for comparison among groups. Variables following skewed distribution were presented as median [Q1, Q3]. Mann-Whitney test was used for comparison between two groups. Kruskal-Wallis test followed by Dunn's multiple comparison test was used for comparison among groups. A *P* < 0.05 was considered as statistical difference.

## Results

### IL-24 Was Decreasingly Expressed in Colorectal Adenocarcinoma

**Figure 1:**
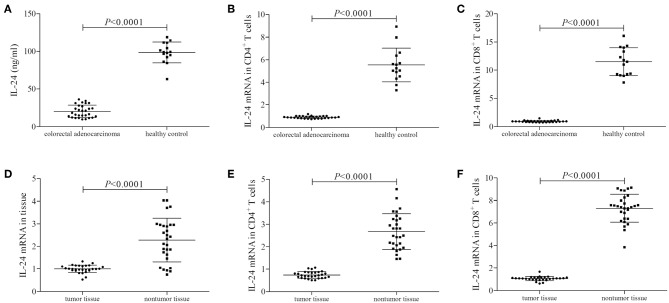
IL-24 expression profile in colorectal adenocarcinoma patients. Plasma IL-24 concentration was measured by ELISA in all enrolled subjects (29 of colorectal adenocarcinoma patients and 15 of healthy controls). IL-24 mRNA relative level was measured by real-time PCR in tumor tissue, peripheral and tumor-infiltrating CD4^+^ and CD8^+^ T cells. **(A)** IL-24 expression in the plasma was reduced in colorectal adenocarcinoma patients. **(B)** IL-24 mRNA expression was down-regulated in peripheral CD4^+^ T cells from colorectal adenocarcinoma patients. **(C)** IL-24 mRNA expression was also down-regulated in peripheral CD8^+^ T cells from colorectal adenocarcinoma patients. **(D)** IL-24 mRNA expression was decreased in colorectal adenocarcinoma tissue. **(E)** IL-24 mRNA expression was down-regulated in tumor-infiltrating CD4^+^ T cells from colorectal adenocarcinoma tissue. **(F)** IL-24 mRNA expression was down-regulated in tumor-infiltrating CD8^+^ T cells from colorectal adenocarcinoma tissue. Individual level of each subject was shown. Student's *t*-test, One-way ANOVA, or Mann-Whitney test was used for comparison.

We firstly screened the protein and mRNA expression profile of IL-24 in colorectal adenocarcinoma patients. IL-24 concentration in the plasma was measured by ELISA. Plasma IL-24 expression was robustly reduced in colorectal adenocarcinoma patients when compared with healthy controls (20.21 ± 8.15 ng/ml vs. 98.51 ± 18.94 ng/ml; Student's *t*-test, *P* < 0.0001, Figure 1A). However, there were no significant differences of IL-24 concentration among colorectal adenocarcinoma patients with different tumor site (One-way ANOVA, *P* > 0.05), different differentiation (One-way ANOVA, *P* > 0.05), or in different TNM stages (One-way ANOVA, *P* > 0.05). IL-24 mRNA relative level was also semi-quantified in peripheral T cells by real-time PCR. IL-24 mRNA expression was also notably down-regulated in both peripheral CD4^+^ and CD8^+^ T cells from colorectal adenocarcinoma patients when compared with those from healthy individuals (Student's *t*-tests, all *P* < 0.0001, Figures 1B,C). Furthermore, IL-24 mRNA was also investigated in tumor tissue and tumor-infiltrating T cells from colorectal adenocarcinoma patients. IL-24 mRNA relative level in tumor tissue was decreased when compared with non-tumor tissue (Mann-Whitney test, *P* < 0.0001, Figure 1D). Similarly, IL-24 mRNA expression in tumor-infiltrating CD4^+^ and CD8^+^ T cells was also remarkably reduced when compared with those from non-tumor tissue (Mann-Whitney tests, all *P* < 0.0001, Figures 1E,F). However, there were no significant differences of peripheral or tissue resident IL-24 mRNA relative level among colorectal adenocarcinoma patients with different tumor site (Kruskal-Wallis test, *P* > 0.05), different differentiation (Kruskal-Wallis test, *P* > 0.05), or in different TNM stages (Kruskal-Wallis test, *P* > 0.05).

### IL-20R1 and IL-20R2 Did Not Significantly Changed in Colorectal Adenocarcinoma

**Figure 2:**
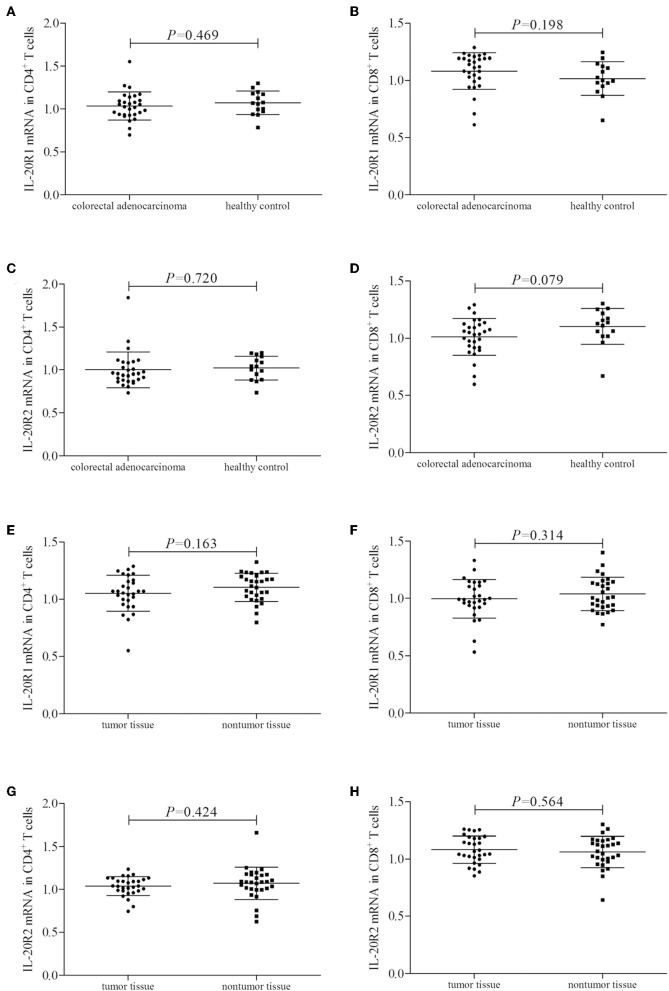
IL-24 receptor component expression in colorectal adenocarcinoma patients. IL-20R1, IL-20R2, and IL-22R2 mRNA relative level was semi-quantified in peripheral and tumor-infiltrating CD4^+^ and CD8^+^ T cells from all enrolled subjects (29 of colorectal adenocarcinoma patients and 15 of healthy controls). **(A)** IL-20R1 mRNA in peripheral CD4^+^ T cells. **(B)** IL-20R1 mRNA in peripheral CD8^+^ T cells. **(C)** IL-20R2 mRNA in peripheral CD4^+^ T cells. **(D)** IL-20R2 mRNA in peripheral CD8^+^ T cells. **(E)** IL-20R1 mRNA in tumor-infiltrating CD4^+^ T cells. **(F)** IL-20R1 mRNA in tumor-infiltrating CD8^+^ T cells. **(G)** IL-20R2 mRNA in tumor-infiltrating CD4^+^ T cells. **(H)** IL-20R2 mRNA in tumor-infiltrating CD8^+^ T cells. IL-20R1 and IL-20R2 mRNA was comparable in peripheral and tumor-infiltrating CD4^+^ and CD8^+^ T cells between colorectal adenocarcinoma patients and healthy individuals, and between tumor tissue and non-tumor tissue. Individual level of each subject was shown. Student's *t*-test was used for comparison.

mRNA expression corresponding to the component of IL-24 receptor, including IL-20R1, IL-20R2, and IL-22R1, was semi-quantified in peripheral and tumor-infiltrating CD4^+^ and CD8^+^ T cells. IL-20R1 mRNA relative level was comparable in peripheral CD4^+^ and CD8^+^ T cells between colorectal adenocarcinoma patients and healthy individuals (Student's *t*-tests, *P* < 0.05, Figures 2A,B). IL-20R2 mRNA relative level was also comparable in peripheral T cells between two groups (Student's *t*-tests, *P* < 0.05, Figures 2C,D). IL-20R1/IL-20R2 expression presented similar trends in tumor-infiltrating T cells. IL-20R1 and IL-20R2 mRNA was also comparable in tumor-infiltrating CD4^+^ and CD8^+^ T cells between colorectal adenocarcinoma tissue and non-tumor tissue (Student's *t*-tests, *P* < 0.05, Figures 2E–H). However, IL-22R1 mRNA was undetectable in either CD4^+^ or CD8^+^ T cells.

### High Concentration of IL-24 Stimulation Enhanced CD4^+^ T Cell Activity in Colorectal Adenocarcinoma

**Figure 3:**
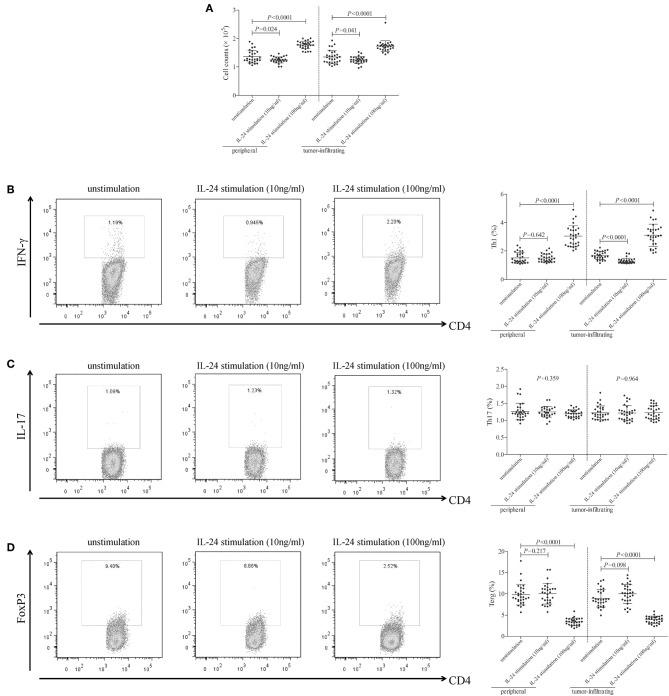
Influence of recombinant IL-24 stimulation on peripheral and tumor-infiltrating CD4^+^ T cell activity in colorectal adenocarcinoma. 10^5^ of purified CD4^+^ T cells from colorectal adenocarcinoma patients (*n* = 29) were stimulated with low concentration (10 ng/ml) or high concentration (100 ng/ml) of recombinant human IL-24 for 24 h. **(A)** Cellular proliferation was measured by CCK-8, and was compared among groups (One-way ANOVA, *P* < 0.0001). **(B)** Representative flow dots of CD4^+^IFN-γ^+^ Th1 cells were shown in unstimulated, 10 ng/ml of IL-24 stimulated, and 100 ng/ml of IL-24 stimulated cells. Percentage of Th1 cells was compared among groups (One-way ANOVA, *P* < 0.0001). **(C)** Representative flow dots of CD4^+^IL-17^+^ Th17 cells were shown in unstimulated, 10 ng/ml of IL-24 stimulated, and 100 ng/ml of IL-24 stimulated cells. Percentage of Th17 cells was compared among groups (One-way ANOVA, *P* > 0.05). **(D)** Representative flow dots of CD4^+^FoxP3 ^+^ Treg were shown in unstimulated, 10 ng/ml of IL-24 stimulated, and 100 ng/ml of IL-24 stimulated cells (One-way ANOVA, *P* < 0.0001). Percentage of Treg was compared among groups. Individual level of each subject was shown. One-way ANOVA and Tukey test for multiple comparison was used for comparison.

10^5^ of peripheral or tumor-infiltrating CD4^+^ T cells from colorectal adenocarcinoma patients were stimulated with low concentration (10 ng/ml) or high concentration (100 ng/ml) of recombinant human IL-24 for 24 h. CCK-8 results showed that 10 ng/ml of IL-24 stimulation slightly inhibited both peripheral and tumor-infiltrating CD4^+^ T cells proliferation (Tukey tests, *P* = 0.024 and *P* = 0.041, respectively, Figure 3A). In contrast, 100 ng/ml of IL-24 stimulation significantly promoted CD4^+^ T cells proliferation (Tukey tests, *P* < 0.0001, Figure 3A). The percentage of Th1 (CD4^+^IFN-γ^+^), Th17 (CD4^+^IL-17^+^), and regulatory T cells (Treg, CD4^+^FoxP3^+^) was assessed by flow cytometry. The gating strategy for CD4^+^ T cells was shown in Figure S1. The representative flow dots was shown in Figures 3B–D, respectively. Low concentration of IL-24 stimulation did not affect the peripheral Th1 percentage (Tukey test, *P* = 0.642, Figure 3B), however, significantly down-regulated tumor-infiltrating Th1 percentage (1.30 ± 0.19% vs. 1.67 ± 0.28%, Tukey test, *P* < 0.0001, Figure 3B). In contrast, high concentration of IL-24 robustly increased both peripheral and tumor-infiltrating Th1 frequency (Tukey tests, *P* < 0.0001, Figure 3B). However, IL-24 did not influence either peripheral or tumor-infiltrating Th17 percentage in either low or high concentration manner (One-way ANOVA, *P* > 0.05, Figure 3C). Furthermore, low concentration of IL-24 slightly up-regulated tumor-infiltrating Treg frequency, but this difference failed to achieve statistical significance (10.01 ± 2.24% vs. 8.97 ± 2.08%, Tukey test, *P* = 0.098, Figure 3D). However, low concentration of IL-24 did not affect peripheral Treg frequency (Tukey test, *P* = 0.217, Figure 3D). Importantly, high concentration of IL-24 remarkably reduced both peripheral and tumor-infiltrating Treg frequency (Tukey tests, all *P* < 0.0001, Figure 3D).

**Figure 4:**
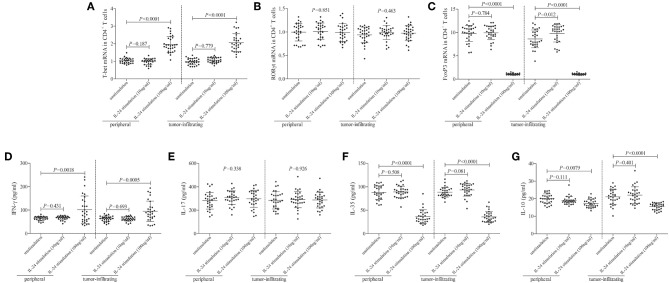
Influence of recombinant IL-24 stimulation on transcriptional factor and cytokine production of peripheral and tumor-infiltrating CD4^+^ T cells in colorectal adenocarcinoma. 10^5^ of purified CD4^+^ T cells from colorectal adenocarcinoma patients (*n* = 29) were stimulated with low concentration (10 ng/ml) or high concentration (100 ng/ml) of recombinant human IL-24 for 24 h. mRNA expression of transcriptional factors, including **(A)** T-bet (One-way ANOVA, *P* < 0.0001), **(B)** ROR-γt (One-way ANOVA, *P* > 0.05), and **(C)** FoxP3 (One-way ANOVA, *P* < 0.0001), were semi-quantified by real-time PCR, and were compared among groups. Expression of cytokines in cultured supernatants, including **(D)** IFN-γ (Kruskal-Wallis test, *P* < 0.05), **(E)** IL-17 (One-way ANOVA, *P* > 0.05), **(F)** IL-35 (One-way ANOVA, *P* < 0.0001), and **(G)** IL-10 (One-way ANOVA, *P* < 0.001), was measured by ELISA, and were compared among groups. Individual level of each subject was shown. One-way ANOVA, Tukey test for multiple comparison, Kruskal-Wallis test, Dunn's multiple comparison test was used for comparison.

mRNA expression of transcriptional factors of CD4^+^ T cells, including T-bet (Th1 transcriptional factor), retinoic acid receptor-related orphan receptor-γt (ROR-γt, Th17 transcriptional factor), and FoxP3 (Treg transcriptional factor), was semi-quantified by real-time PCR. Low concentration of IL-24 did not affect T-bet mRNA relative level in either peripheral or tumor-infiltrating CD4^+^ T cells (Tukey tests, *P* > 0.05, Figure 4A). In contrast, high concentration of IL-24 robustly elevated T-bet mRNA relative level in CD4^+^ T cells (Tukey tests, *P* < 0.0001, Figure 4A). IL-24 stimulation did not influence ROR-γt mRNA expression in CD4^+^ T cells (One-way ANOVA, *P* > 0.05, Figure 4B), which were similar to the trends of Th17 percentage. Moreover, low concentration of IL-24 promoted FoxP3 mRNA expression in tumor-infiltrating CD4^+^ T cells (Tukey test, *P* = 0.012, Figure 4C). However, high concentration of IL-24 notably down-regulated FoxP3 mRNA relative level in both peripheral and tumor-infiltrating CD4^+^ T cells (Tukey tests, *P* < 0.0001, Figure 4C). Cytokine production in the cultured supernatants was measured by ELISA. Th1-secreting cytokine IFN-γ was comparable between unstimulated and 10 ng/ml of IL-24 stimulated CD4^+^ T cells (Dunn's multiple comparison tests, *P* > 0.05, Figure 4D). One hundred Nanograms per milliliter of IL-24 stimulation enhanced IFN-γ expression in both peripheral and tumor-infiltrating CD4^+^ T cells (Dunn's multiple comparison tests, *P* < 0.0001, Figure 4D). IL-24 stimulation did not affect Th17-secreting cytokine IL-17 production by CD4^+^ T cells (One-way ANOVA, *P* > 0.05, Figure 4E). Expression of Treg-secreting cytokine, IL-35 and IL-10, presented similar trends of Tregs frequency and FoxP3 mRNA. Low concentration of IL-24 did not influence IL-35 and IL-10 production by CD4^+^ T cells (Tukey tests, *P* > 0.05, Figures 4F,G), while high concentration of IL-24 dampened IL-35 and IL-10 expression in cultured CD4^+^ T cells (Tukey tests, *P* < 0.0001, Figures 4F,G).

### High Concentration of IL-24 Promoted CD8^+^ T Cell Function in Colorectal Adenocarcinoma

**Figure 5:**
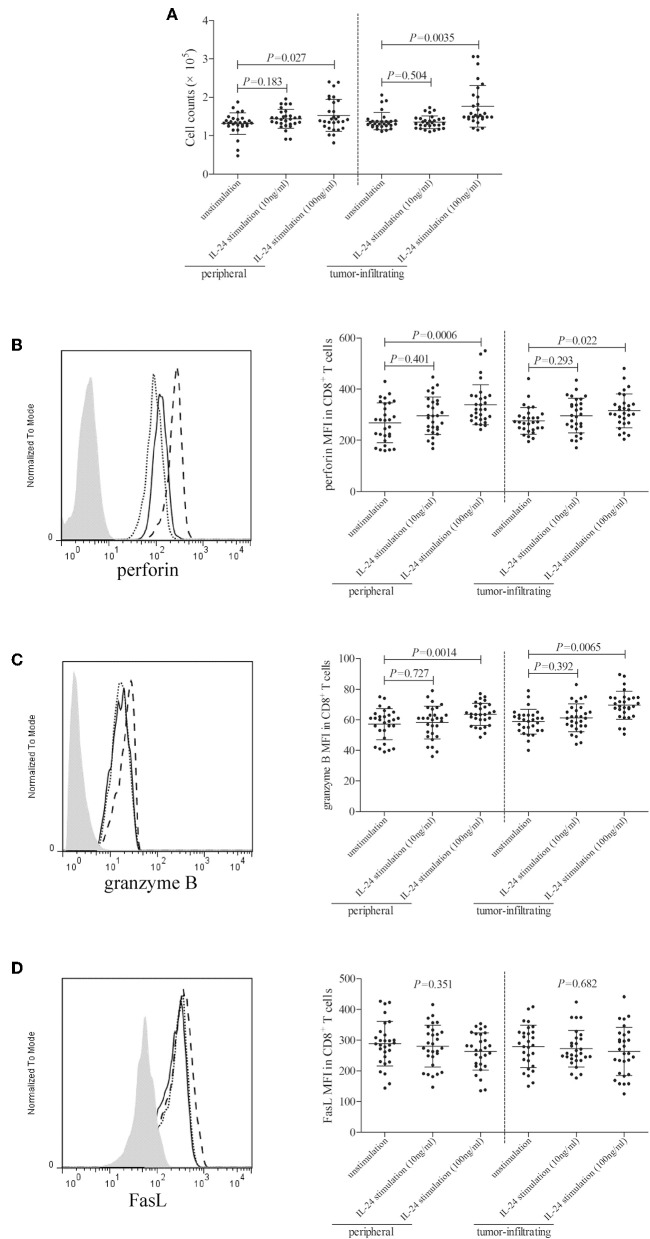
Influence of recombinant IL-24 stimulation on peripheral and tumor-infiltrating CD8^+^ T cell activity in colorectal adenocarcinoma. 10^5^ of purified CD8^+^ T cells from colorectal adenocarcinoma patients (*n* = 29) were stimulated with low concentration (10 ng/ml) or high concentration (100 ng/ml) of recombinant human IL-24 for 24 h. **(A)** Cellular proliferation was measured by CCK-8, and was compared among groups (One-way ANOVA, *P* < 0.05). **(B)** perforin, **(C)** granzyme B, and **(D)** FasL expression was assessed by flow cytometry, and representative histograms were shown. MFI corresponding to **(B)** perforin (One-way ANOVA, *P* < 0.01), **(C)** granzyme B (One-way ANOVA, *P* < 0.05), and **(D)** FasL (One-way ANOVA, *P* > 0.05) was compared among groups. Individual level of each subject was shown. One-way ANOVA and Tukey test for multiple comparison was used for comparison.

10^5^ of peripheral or tumor-infiltrating CD8^+^ T cells from colorectal adenocarcinoma patients were stimulated with low concentration (10 ng/ml) or high concentration (100 ng/ml) of recombinant human IL-24 for 24 h. CCK-8 results showed that 10 ng/ml of IL-24 stimulation did not affect CD8^+^ T cells proliferation (Tukey tests, *P* > 0.05, Figure 5A). However, 100 ng/ml of IL-24 stimulation significantly increased CD4^+^ T cells proliferation (Tukey tests, all *P* < 0.05, Figure 5A). Cytotoxic molecules in CD8^+^ T cells, including perforin, granzyme B, and FasL, were assessed by flow cytometry. The gating strategy for CD8^+^ T cells was shown in Figure S2.The representative histograms were shown in Figures 5B–D, respectively. Perforin and granzyme B was expressed in almost all CD8^+^ T cells. Mean Fluorescence Intensity (MFI) corresponding to perforin and granzyme B was then analyzed. As shown in Figures 5B,C, low concentration of IL-24 did not affect either perforin or granzyme B expression in CD8^+^ T cells (Tukey tests, *P* > 0.05), while high concentration of IL-24 promoted perforin and granzyme B expression in both peripheral and tumor-infiltrating CD8^+^ T cells (Tukey tests, *P* < 0.05). Furthermore, ~60% of CD8^+^ T cells expressed FasL. However, there were no significant differences of either FasL^+^ cell frequency or FasL MFI among unstimulated and IL-24 stimulated CD8^+^ T cells (Tukey tests, *P* > 0.05, Figure 5D).

**Figure 6:**
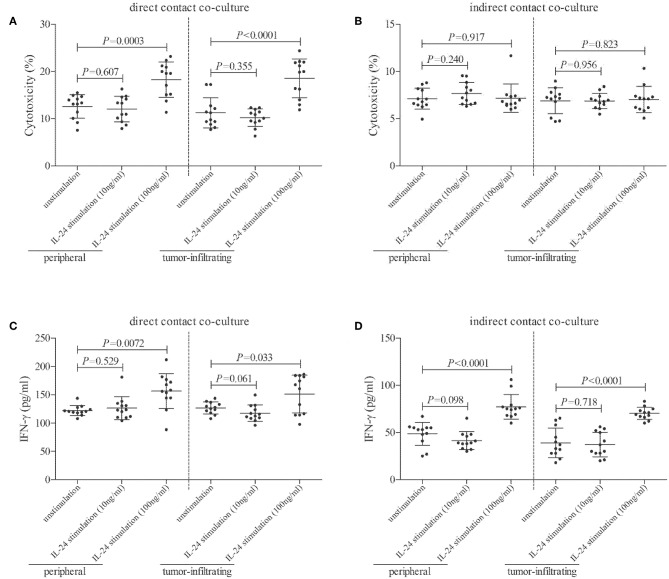
Influence of recombinant IL-24 stimulation on cytolytic and non-cytolytic function of CD8^+^ T cells in colorectal adenocarcinoma. CD8^+^ T cells were purified from HLA-A2 restricted colorectal adenocarcinoma patients (*n* = 12), and were stimulated low concentration (10 ng/ml) or high concentration (100 ng/ml) of recombinant human IL-24 for 24 h. 5 × 10^4^ of IL-24 stimulated CD8^+^ T cells were co-cultured in direct contact and in parallel in indirect contact system with 2.5 × 10^5^ of colorectal adenocarcinoma cell line CACO-2 for 48 h. Cytotoxicity of target CACO-2 cells was calculated by measurement of LDH expression in the cultured supernatants. IFN-γ and TNF-α expression in the cultured supernatants was measured by ELISA. **(A)** Cytotoxicity of target CACO-2 cells in direct contact co-culture system (One-way ANOVA, *P* < 0.01). **(B)** Cytotoxicity of target CACO-2 cells in indirect contact co-culture system (One-way ANOVA, *P* > 0.05). **(C)** IFN-γ expression in direct contact co-culture system (One-way ANOVA, *P* < 0.001). **(D)** IFN-γ expression in indirect contact co-culture system (One-way ANOVA, *P* < 0.0001). Individual level of each subject was shown. One-way ANOVA and Tukey test for multiple comparison was used for comparison.

CD8^+^ T cells, which were purified from twelve HLA-A2 restricted colorectal adenocarcinoma patients, were stimulated with recombinant IL-24 for 24 h, and were then co-cultured with CACO-2 cells (effector: target = 1: 5) in both direct contact and indirect contact manner. Supernatants were harvested 48 h post co-culture. In direct contact co-culture system, high concentration of IL-24 (100 ng/ml), but not low concentration, stimulated CD8^+^ T cells induced higher percentage of target CACO-2 cell death (Tukey tests, *P* < 0.001, Figure 6A). However, neither high nor low concentration of IL-24 promoted CD8^+^ T cells-induced cell death in indirect contact co-culture system (Tukey tests, *P* > 0.05, Figure 6B). Furthermore, high concentration of IL-24, but not low concentration, treated CD8^+^ T cells enhanced IFN-γ production in both direct and indirect contact co-culture system (Tukey tests, *P* < 0.05, Figures 6C,D).

## Discussion

In the current study, IL-24 was decreasingly expressed in both peripheral bloods and cancer tissues in colorectal adenocarcinoma, but did not correlate with either histological differentiation or TNM staging. IL-24 receptor component, IL-20R1 and IL-20R2, was comparable in CD4^+^/CD8^+^ T cells between normal controls and colorectal adenocarcinoma. However, IL-22R2 was undetectable in T cells. Furthermore, low concentration (10 ng/ml) of IL-24 stimulation dampened CD4^+^ T cell proliferation, but not affected bioactivity of either CD4^+^ or CD8^+^ T cells. In contrast, high concentration (100 ng/ml) of IL-24 stimulation promoted both CD4^+^ and CD8^+^ T cell function, which presented as increase of Th1/Th17 cells and elevation of cytolytic and non-cytolytic activity of peripheral and tumor-infiltrating CD8^+^ T cells. The current results suggested an important immunomodulatory function of IL-24 to T cells in a dose-dependent manner in colorectal adenocarcinoma.

It was well accepted that IL-24 mediated cancer cell-specific death and apoptosis *via* multiple signaling pathways (21–23). Significantly lower IL-24 expression predicted poorer prognosis in lung adenocarcinoma (24), breast cancer (25), head and neck squamous cell carcinoma (26), and lymphoma (27, 28). IL-24^high^ adenocarcinoma patients showed a notably higher incidence of apoptotic tumor cell death, and displayed favorable post-therapy prognosis as compared with IL-24^low^ patients (24). Similarly, colorectal cancer tissue revealed significantly lower IL-24 level, which was associated with 5-year survival rate (17). This was consistent with our present findings, which indicated a robust decline of IL-24 in colorectal adenocarcinoma tissues, as well as in peripheral and tumor-infiltrating T cells from colorectal adenocarcinoma patients. However, down-regulation of IL-24 level did not correlate with either histological differentiation or TNM staging, which diversely reported previously (15). This might partly due to the differences in detection and semi-quantification methods. Collectively, reduced expression of circulating and tissue-resident IL-24 might contribute to pathogenesis and progression of colorectal adenocarcinoma.

Controversy remains as to the regulatory activity of IL-24 to immune systems in infectious diseases. Parasite-specific CD4^+^ and CD8^+^ T cells expressing IL-19 and IL-24 was significantly increased in human lymphatic filariasis (29), and the elevation of IL-19 and IL-24 in turn modulated CD4^+^ and CD8^+^ T cell function during filarial infections, which presented as down-regulation of Th1/Tc1 and Th17/Tc17 cells (30). Similarly, the increased expression of IL-19 and IL-24 in active pulmonary tuberculosis patients also mediated decreased expression of Th1/Tc1 and Th17/Tc17 cytokine in CD4^+^ and CD8^+^ T cells (31). In contrast, IL-24 stimulated neutrophils to produce IFN-γ and IL-12, subsequently activating CD8^+^ T cells during Salmonella typhimurium infection both *in vitro* and *in vivo* (32). However, IL-24 was originally identified as a tumor suppressor cytokine. Thus, modulation of IL-24 to immune cells from cancer patients might be completely different. Adenovirus-mediated IL-24 vaccinated mice showed increased production of IFN-γ and higher proliferative activity in spleonocytes, which was mainly elevated in CD8^+^ T cells, but not CD4^+^ T cells (33). Importantly, IL-24 also enhanced IFN-γ secretion by T cells and promoted cytotoxicity of CD8^+^ T cells in a colon cancer mouse model (34). But the role of direct regulatory activity of IL-24 to tumor-infiltrating T cells in colorectal adenocarcinoma patients still needed further elucidation.

We firstly investigated the expression profile of IL-24 receptor in T cells. There were no significant differences of IL-20R1 and IL-20R2 in peripheral and tissue-resident T cells between healthy individuals and colorectal adenocarcinoma patients, as well as between normal and tumor tissues. However, IL-22R1 could not be detected in either CD4^+^ or CD8^+^ T cells. This was consistent with the previous findings, which demonstrated that IL-22 receptor was strictly expressed on the tissue but absent on immune cells (35). Thus, signaling through IL-24 in regulation of T cells was dependent on the expression of IL-20R1/IL-20R2 heterodimeric complex, which was also the receptor for IL-20 (11). Furthermore, since anti-tumor property of IL-24 was dose-dependent, we also chose two different concentration (10 ng/ml as low concentration and 100 ng/ml as high concentration) of recombinant IL-24 for stimulation based on the plasma IL-24 level in colorectal adenocarcinoma patients and healthy individuals. Low concentration of IL-24 inhibited CD4^+^ T cell proliferation and dampened tumor-infiltrating Th1 response. In contrast, high concentration of IL-24 robustly promoted CD4^+^ T cell proliferation, enhanced Th1 and Th17 response, and inhibited Treg response in colorectal adenocarcinoma patients. This was similar to the *in vivo* findings that administration of 50 μg recombinant IL-24 promoted CD4^+^ T cells response, especially increased IFN-γ production in colon cancer mouse model (34). This was partly due to the sufficient ligation of high concentration IL-24 to IL-20R1/IL-20R2, which fully activated down-stream signaling pathways in CD4^+^ T cells. However, the current results suggested low percentages of Th1 and Th17 cells in both peripheral and tumor-infiltrating CD4^+^ T cells from colorectal adenocarcinoma patients. This was contrast with the previous reports which showed ~45% of Th1 (36) and 5% of Th17 (37) within colorectal cancer-infiltrating CD4^+^ T cells. The difference might be due to different antibody clones used for staining or variations in geographic location and host genetic background, because several studies on Chinese population also revealed similar and low frequency of Th1 and Th17 cells (38, 39).

CD8^+^ T cells induced tumor rejection *via* both cytolytic (direct target cell cytotoxicity) and non-cytolytic (cytokine production) function (20, 40, 41). There were two independent pathways, perforin/granzyme B pathway and Fas/FasL interaction, which contributed to the cytolytic activity of CD8^+^ T cells (42). Low concentration of IL-24 did not affect the bioactivity of CD8^+^ T cells. However, high concentration of IL-24 also notably increased CD8^+^ T cell proliferation, and elevated perforin/granzye B, but not FasL expression in CD8^+^ T cells from peripheral and tumor-infiltrating CD8^+^ T cells from colorectal adenocarcinoma patients. This indicated that high concentration of IL-24 mainly influenced perforin/granzyme B pathway for cytolytic function of CD8^+^ T cells in colorectal adenocarcinoma. The cytolytic and non-cytolytic function of CD8^+^ T cells was also distinguished in direct contact and indirect contact co-culture system. High concentration of IL-24 promoted both peripheral and tumor-infiltrating CD8^+^ T cells-induced cytotoxicity only in direct contact co-culture system, while IFN-γ production was elevated in both systems. The current results demonstrated that non-cytolytic activity of CD8^+^ T cells was insufficient for colorectal ademocarcinoma rejection, although IL-24 promoted both cytolytic and non-cytolytic function of CD8^+^ T cells.

The limitation of the current study was that the majority of enrolled patients was in stage I and stage II (26 out of 29). This was due to the fact that we need to analyze the TILs from colorectal carcinoma patients who underwent surgery. However, most patients in stage IV already lost the opportunity for operation, while biopsy samples were insufficient for TILs isolation. Thus, no patients in stage IV was enrolled.

In conclusion, down-regulation of circulating and tissue-resident IL-24 in colorectal adenocarcinoma was inadequate for developing anti-tumor activity. IL-24 regulated T cell function in a dose-dependent manner. High-concentration of IL-24 promoted peripheral and tumor-infiltrating CD4^+^ and CD8^+^ T cell function, which provided novel therapeutic approaches to colorectal adenocarcinoma.

## Data Availability Statement

The datasets generated for this study are available on request to the corresponding author.

## Ethics Statement

The studies involving human participants were reviewed and approved by the Ethics Committee of The First Hospital of Jilin University. The patients/participants provided their written informed consent to participate in this study.

## Author Contributions

YZ and YL performed the study. YZ and YX enrolled patients. YZ, YL, and YX analyzed the data and prepared the manuscript. YX supervised the study.

### Conflict of Interest

The authors declare that the research was conducted in the absence of any commercial or financial relationships that could be construed as a potential conflict of interest.
