# Supplementary material for: Interleukin-24 Regulates T Cell Activity in Patients With Colorectal Adenocarcinoma
Source: Front Oncol. 2019 Dec 10;9:1401. doi: 10.3389/fonc.2019.01401 (PMC6915036; doi:10.3389/fonc.2019.01401)
Supplement: Supplementary file 1 [file Data_Sheet_1.PDF]

Interleukin-24 regulates T cell activity in patients with colorectal adenocarcinoma

Running head: IL-24 regulates T cells in CRC

Yang Zhang [1], Ye Liu [2], Yuechao Xu [1]*

1. Department of Gastrointestinal Surgery, The First Hospital of Jilin University, Changchun, Jilin Province, 130021, China

2. Intensive Care Unit, 964th Hospital of PLA, Changchun, Jilin Province, 130062, China

Corresponding author: **Yuechao Xu**

E-mail: forever981024@vip.qq.com or forever981024@tom.com

Table S1. Variables following normal and skewed distribution

Variables following normal distribution	Variables following skewed distribution
IL-24 expression in the plasma	IL-24 mRNA in tumor tissue
IL-24 mRNA in peripheral $CD4^+$ T cells	IL-24 mRNA in tumor-infiltrating $CD4^+$ T cells
IL-24 mRNA in peripheral $CD8^+$ T cells	IL-24 mRNA in tumor-infiltrating $CD8^+$ T cells
IL-20R1 mRNA in $CD4^+$ T cells	IFN-γ production by $CD4^+$ T cells
IL-20R2 mRNA in $CD8^+$ T cells	
Th1, Th17, and Treg percentage in $CD4^+$ T cells	
T-bet, ROR-γt, and FoxP3 mRNA in $CD4^+$ T cells	
IL-17, IL-35, and IL-10 production by $CD4^+$ T cells	
Perforin, granzyme B, and FasL mRNA in $CD8^+$ T cells	
Percentage of target cell death	
IFN-γ production in co-culture systems	

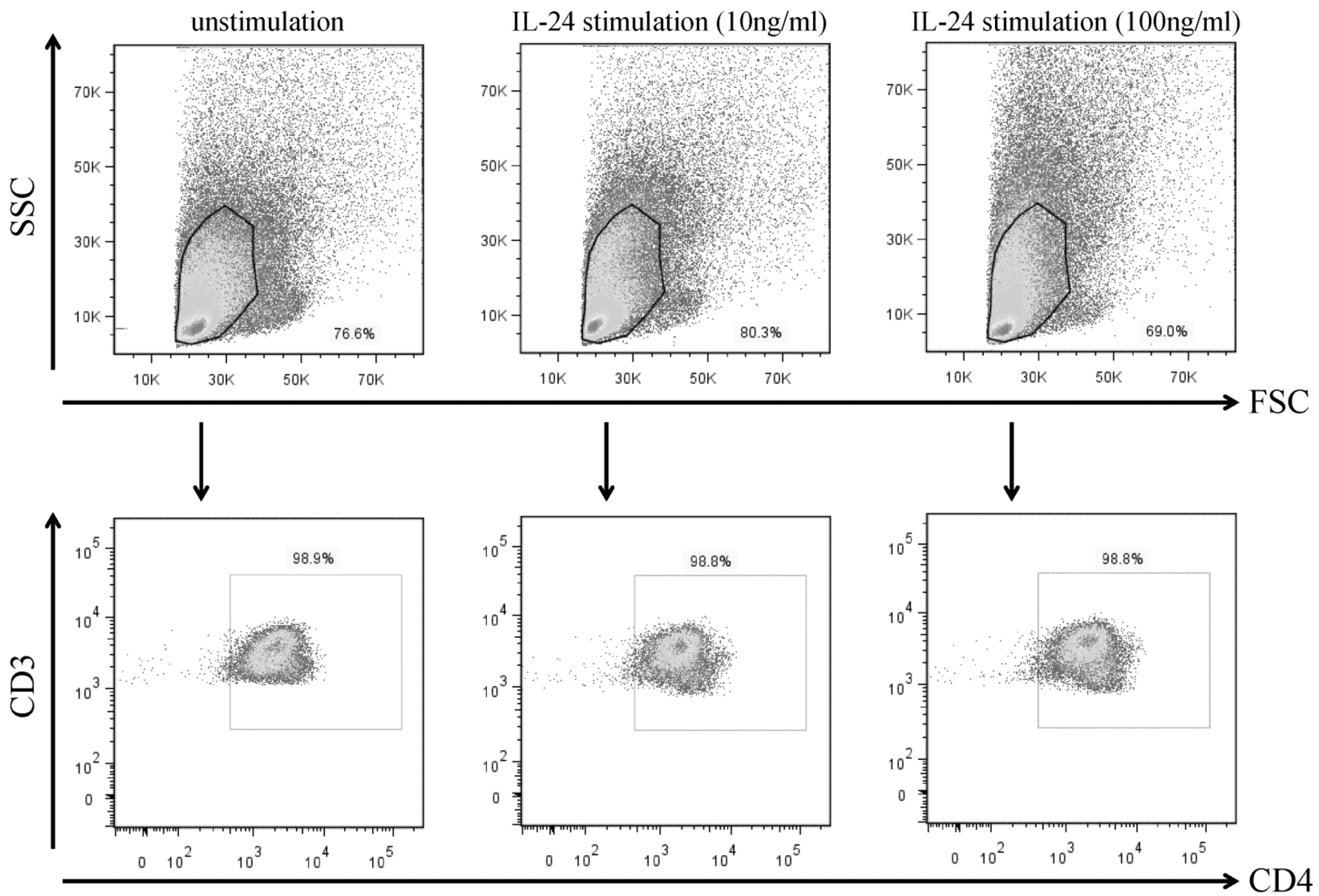

Figure S1. Gating strategy for $CD4^{+}$ T cells with and without IL-24 stimulation.

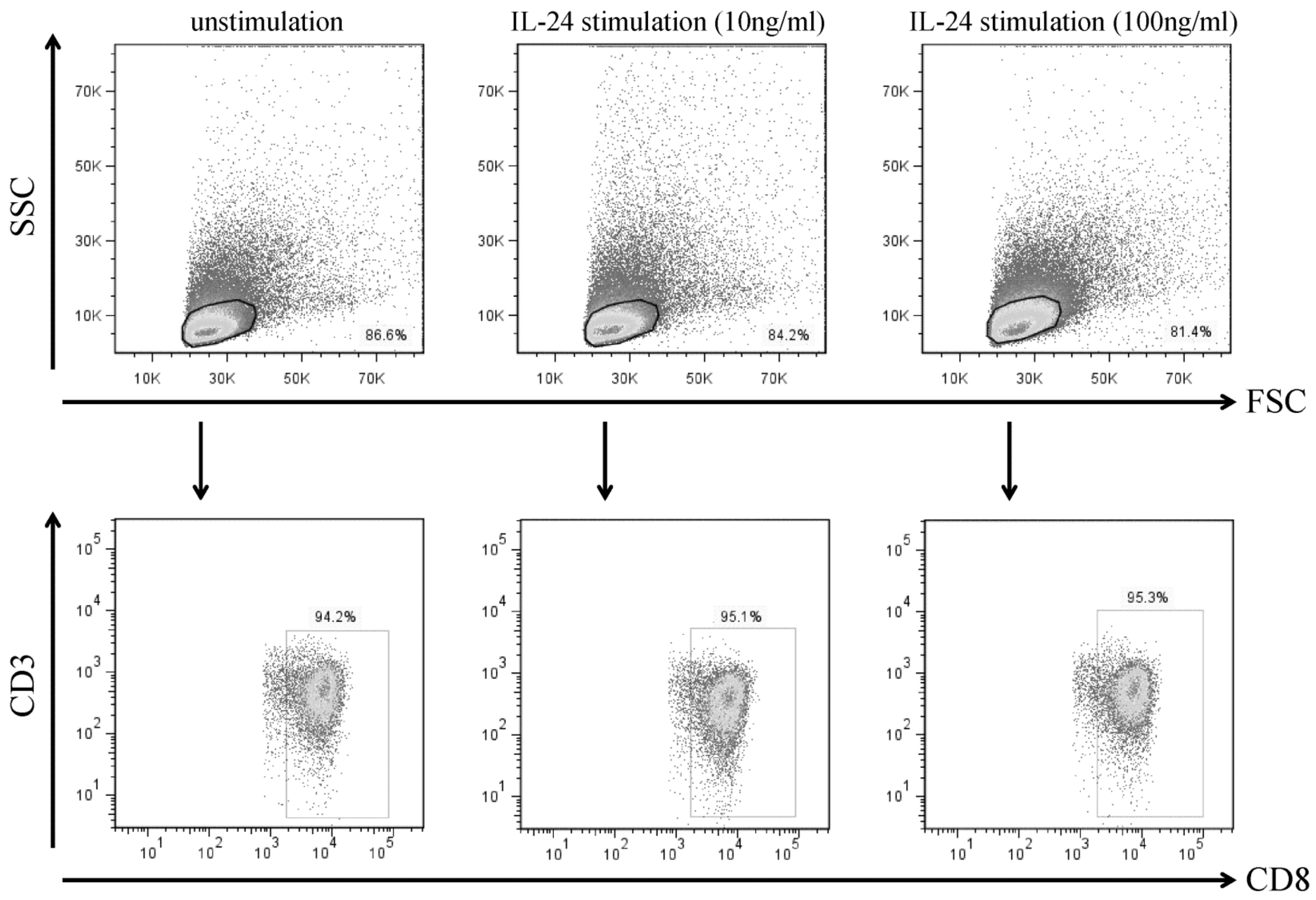

Figure S2. Gating strategy for $CD8^+$ T cells with and without IL-24 stimulation.
